# Extracts of medicinal plants with natural deep eutectic solvents: enhanced antimicrobial activity and low genotoxicity

**DOI:** 10.1186/s13065-020-00726-x

**Published:** 2020-12-11

**Authors:** Tsvetinka Grozdanova, Boryana Trusheva, Kalina Alipieva, Milena Popova, Lyudmila Dimitrova, Hristo Najdenski, Maya M. Zaharieva, Yana Ilieva, Bela Vasileva, George Miloshev, Milena Georgieva, Vassya Bankova

**Affiliations:** 1 Institute of Organic Chemistry With Centre of Phytochemistry, Bulgarian Academy of Sciences, Acad. G. Bonchev Str., Bl. 9, 1113 Sofia, Bulgaria; 2 The Stephan Angeloff Institute of Microbiology, Bulgarian Academy of Sciences, Acad. G. Bonchev Str., Bl. 26, 1113 Sofia, Bulgaria; 3 Institute of Molecular Biology “Roumen Tsanev”, Bulgarian Academy of Sciences, Acad. G. Bonchev Str., Bl. 21, 1113 Sofia, Bulgaria

**Keywords:** Green extraction, Natural deep eutectic solvents, *Sideritis scardica*, *Plantago major*, Antimicrobial activity, Genotoxicity

## Abstract

Natural deep eutectic solvents (NADES) are a new alternative to toxic organic solvents. Their constituents are primary metabolites, non-toxic, biocompatible and sustainable. In this study four selected NADES were applied for the extraction of two medicinal plants: *Sideritis scardica*, and *Plantago major* as an alternative to water-alcohol mixtures, and the antimicrobial and genotoxic potential of the extracts were studied. The extraction efficiency was evaluated by measuring the extracted total phenolics, and total flavonoids. Best extraction results for total phenolics for the studied plants were obtained with choline chloride-glucose 5:2 plus 30% water; but surprisingly these extracts were inactive against all tested microorganisms. Extracts with citric acid-1,2-propanediol 1:4 and choline chloride-glycerol 1:2 showed good activity against *S. pyogenes*, *E. coli*, *S. aureus*, and *C. albicans*. Low genotoxicity and cytotoxicity were observed for all four NADES and the extracts with antimicrobial activity. Our results confirm the potential of NADESs for extraction of bioactive constituents of medicinal plants and further suggest that NADES can improve the effects of bioactive extracts. Further studies are needed to clarify the influence of the studied NADES on the bioactivity of dissolved substances, and the possibility to use such extracts in the pharmaceutical and food industry.
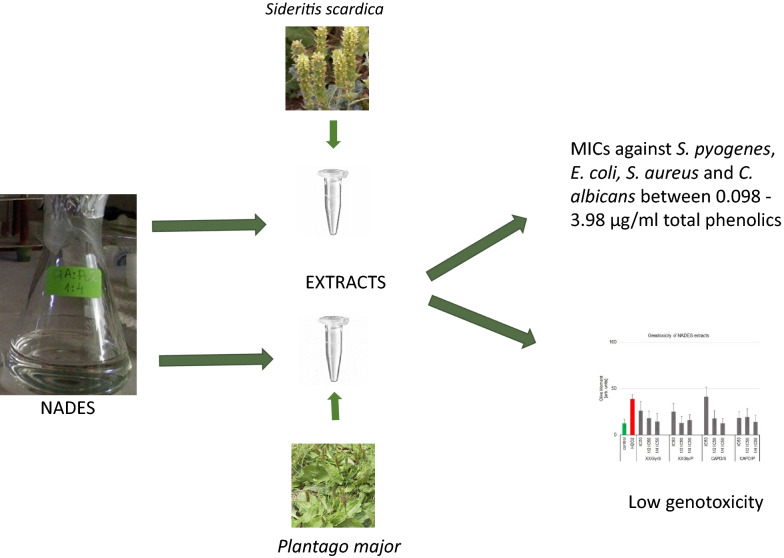

## Introduction

One of the most important aims of green chemistry has been to find green solvents for extraction of bioactive compounds from natural sources in order to replace the currently used hazardous organic solvents. One of the eco-friendly alternatives are deep eutectic solvents (DES) and particularly the natural deep eutectic solvents (NADES). DES are mixtures of organic compounds that have melting points lower than those of the individual components of the mixture and are liquid at ambient temperature. In the case of NADES, the constituents of the eutectic mixture are natural compounds: primary metabolites, which are easily available, non-toxic, biocompatible and sustainable [1]. NADES have low vapor pressure, an advantage with respect to environmental and human health protection. This property, however, poses a serious problem to the recovery of the active ingredients from the extract. Recently, several studies have demonstrated that NADESs retained or even improved the biological activity of dissolved substances [2, 3]. Therefore, the NADES could function as an active ingredient, and the extract could be directly used as part of cosmetic or pharmaceutical formulations, bypassing the difficulties of solute recovery. The aim of the present study was to apply selected NADES for extraction of two popular Bulgarian medicinal plants: the mountain tea *Sideritis scardica* Griesb., and the broadleaf plantain *Plantago major* L. as an alternative to water–alcohol mixtures, and to evaluate the antimicrobial, cytotoxic and genotoxic potential of both NADES solvents and extracts.

## Materials and methods

### Chemicals and reagents

Ethanol (absolute) was obtained from Alkaloid (Skopje, Macedonia). Glycerol and 1,2-propanediol were purchased from Valerus (Sofia, Bulgaria); choline chloride and Nile Red from Sigma Aldrich (Switzerland), citric acid and glucose from Chem-Lab NV (Zedelgem, Belgium) and Fisher Chemical (Loughborough, UK), respectively. The chemicals used in the in vitro cytotoxicity assay were purchased from Sigma® Life Science, (Steinheim, Germany): 3-(4,5-dimethylthiazolyl-2)-2,5-diphenyltetrazolium bromide (MTT, #M2128-1G), ethylenediaminetetraacetic acid (EDTA, #E6635), l-glutamin (#G7513) and Dulbecco’s phosphate buffered saline (PBS, #D8537). Media, enzymes and sera for cultivation CCL-1 cells originated from Capricorn®, Germany: MEM (#MEM-A), horse serum (#HOS-1A), Pen/Strep 100*x*(#PS-B), Trypsin (#TRY-1B10, #TRY-2B10). HCOOH was delivered from Chimspektar OOD (Bulgaria).

### Plant material

Aerial parts of the studied plants were used. *Plantago major* was collected in March 2018 in the valley of River Struma (: 41°56′46.24″ N 23°5′54.23″ E, 212 m a.s.l.), a voucher specimen (No. SOM 1390) has been deposited in the Herbarium of the Institute of Biodiversity and Ecosystem Research, Bulgarian Academy of Sciences (IBER-BAS). *Sideritis scardica* collected in June 2017, was cultivated in the Western Rhodope Mountain (41°57′36.34″ N 23°39′19.32″ E, 1202 m a.s.l.), a voucher specimen has been deposited in the Herbarium of the IBER-BAS, No. SOM 1391. Plant material was collected and identified by Assoc. Prof. Dr. Ina Aneva.

### Preparation of NADES

The NADES were prepared by mixing the components and subsequently stirring in water bath (300 rpm) combined with mild heating at 50 °C until a homogeneous liquid was formed [1].

### Polarity measurements

The NADESs polarity was measured by the solvatochromic dye Nile red [4]. The dye was dissolved in each NADES in the concentration range 0.01–0.1 mM and absorption spectra were recorded. The λ_max_ was used to calculate the molar transition energy ENR, based on the equation: ENR = hc_NA_/λ_max_ = 28,591/λ_max_, (ENR in kcal/mol, λ_max_ in nm).

### Density measurements

The NADESs density was determined as follows: 2 ml of NADES were put in a volumetric flask at 20 °C and the weight of the liquid was measured. The density was calculated using the formula: ρ = m_NADES_/V_NADES_, where ρ is density, g/ml at 20 °C, m_NADES_ − weight, g at 20 °C and V_NADES_ − volume in ml at 20 °C (2 ml). For each solvent the procedure was performed in duplicate.

### Extraction

Air-dried plant material was ground using a coffee mill, the average particle size was 0.75 mm. The extraction was performed in a 2 ml Eppendorf tube with 50 mg of plant material and 1.5 ml solvent in an ultrasound bath (Elmasonic S 30 H), without heating, for 1 h. The mixture was then centrifuged at 13,000 rpm for 40 min and filtered through cotton in a 1 ml volumetric flask. This extract was further used for antimicrobial tests, and analyzed to determine the main groups of bioactive compounds in the extracts. Each extraction procedure was performed in triplicate.

### Quantitative determination of total phenolics and total flavonoids

For measuring those two groups of bioactive compounds, previously reported spectrophotometric methods were used [5]. For blank: solution of respective NADES instead of the test sample was used in analogous procedures. Total phenolics content was estimated using caffeic acid as standard, and total flavonoid content with rutine as standard. Every assay was performed in triplicate.

### Antimicrobial activity

#### Test microorganisms

For antimicrobial activities of extracts and solvents, the following test-microorganisms were used: *Bacillus cereus* ATCC 9634 (American Type Cell Culture Collection, USA), *Escherichia coli* ATCC 35218, *Staphylococcus aureus* ATCC 29213, *Pseudomonas aeruginosa* ATCC 27853, *Listeria monocytogenes* C12, *Salmonella typhimurium* 123, *Streptococcus pyogenes* 10535, *Yersinia enterocolitica* 864 O:3 and the fungus *Candida albicans* 562 from the SAIM-BAS collection.

#### Culture medium and growth conditions

Sterilized Brain Heart Infusion Broth and Agar (BHIB, GM210, resp. BHIA, M1611, HiMedia, India) were used as the cultivation media for all bacteria excepting *S. thyphimurium*, *S. aureus* and *E. coli* growing on Muller Hinton Agar and Broth (MHA, CM0337B, resp. MHB, CM0405B, Thermo Scientific-Oxoid, UK), and *C. albicans* SAIMC 562—on Sabouraud-Glucose agar supplemented with gentamicin (40 μg/ml) (CM0041, Oxoid, Basingstoke, UK). All microorganisms were grown at 37 °C overnight except *B. cereus*, which was cultivated at 30 °C and *Y. enterocolitica*—at 26 °C. All microbiological procedures were performed under sterile conditions into a Class II laminar box (FASTER BH-EN 2003, Ferarra, Italy).

#### Minimal inhibitory (MIC) and bactericidal (MBC) concentrations

The antimicrobial activity was studied by the broth microdilution method according to ISO 20776-1:2006. Briefly, bacterial and fungal inoculums with concentration 105 CFU/ml were added to 96-well plates containing BHIB or MHB loaded with twofold serial dilutions of pure solvents or extracts differing in the concentration of total phenolics. Pure solvents were applied in an equivalent concentration as for testing the antimicrobial activity. Plates were incubated overnight at 37 °C, excepting the plates with *B. cereus* and *Y. enterocolitica*, which were incubated at 30 °C, respectively 26 °C. Gentamicin, penicillin and tetracycline were used as reference antibiotics for bacteria and amphotericin B—for *C. albicans*, following the requirements of EUCAST. Experiments were performed in triplicate. MICs and MBCs were determined as described before [6].

#### Dehydrogenase (DEHA) activity

The DEHA activity of the test microorganisms was assessed by MTT-test (3-(4,5-dimethylthiazolyl-2)-2,5-diphenyltetrazolium bromide, M2128-1G, Sigma-Aldrich). The method is based on the reduction of the MTT dye by the membrane located bacterial enzyme NADH: ubiquinone reductase (H+-translocation) to insoluble formazan crystals. Briefly, the treated and untreated bacterial, respectively fungal cells, were incubated for 2 h with MTT dye in a final concentration of 0.05 mg/ml. An equivalent volume of 5% HCOOH in isopropanol dissolved the formed crystals. Absorption was measured using ELISA reader (BioTek Elx800, USA) at 550 nm (reference 690 nm) against a blank solution.

### In vitro cytotoxicity

#### Cell line and culture conditions

The cell line CCL-1™ (mouse fibroblasts, NCTC clone 929, ATCC—American Type Culture Collection, Manassas, Virginia, USA) recommended in Annex C of ISO 10993-5 (ISO 10993-5:2009 2017) for evaluation of in vitro cytotoxicity was cultured in sterile cell culture flasks and controlled environment (incubator Panasonic MCO-18AC, Japan) at 37 °C, 5% CO_2_ and approx. 95% humidity. The cultivation medium MEM was supplemented with 2 mM l-glutamine, 10% heat-inactivated horse serum, 10^5^ Units/l penicillin G sodium and 100 mg/l streptomycin sulphate. Cells were sub-cultivated at a seeding density of 1 × 10^4^ cells/cm^2^ 1–3 times per week after reaching 80–90% confluence. Sequentially, applied solutions of 0.05% EDTA in PBS (1–2 ml, 5–10 min) and 0.25% (w/v)/0.53 mM trypsin/EDTA (1–2 ml, 5–10 min) were used for the detachment of the cell monolayer and cell separation.

#### MTT test—calculation of IC_50_ and statistics

The MTT test was conducted according to Annex C, ISO 10993-5 [7, 8]. Cells with a density of 1 × 10^5^ ml^−1^ were seeded in 96-well plates (flat bottom, 100 µl/well). For cells to start exponential growth (log phase), plates were incubated for 24 h. After entering the log phase cells were exposed to NADES and their extracts at concentrations ranging between 2 and 0.004% volume fraction for 24 and 72 h. PBS was used as a solvent and 4 wells were used for each treatment. MTT (0.5 mg/ml final concentration) was added to each well, followed by a 2-h incubation at 37 °C. The medium above the cells was removed and 100 μl/well 2-propanol supplemented with 5% formic acid were used to dissolve the formed formazan crystals and as a blank solution. Absorption was measured at 540 nm (reference filter 690) on a microplate reader ELx800 (BioTek Instruments, Inc., United States). The IC_50_ values (inhibitory concentration 50 which reduces vital cells by half) were calculated with a non-linear regression analysis (inhibition dose–response model, variable slope) using the GraphPad Prizm software. Untreated cells were considered as negative control and normalized for 100%.

### Genotoxic activity

#### The method of neutral Comet Assay

The method of Comet Assay was performed under neutral conditions. CCL1 cells—control and treated with increasing concentrations of the tested NADES solvents and extracts for 24 h were mixed with 1.4% low-melting agarose and spread onto already pre-coated with 0.5% normal agarose microscopic slides. The microgels, covered with coverslips to assure equal distribution, were incubated at 4 °C for 10 min. The coverslips were removed after solidification of the microgels. This was followed by a 20-min incubation in a lysis buffer (146 mM NaCl, 30 mM EDTA, pH 8; 10 mM Tris–HCl, pH 8; 0.1% *N*-lauroyl sarcosine). Incubation of the gels 3 × 10 min in 0.5×TBE buffer followed. Slides were electrophoresed in 0.5×TBE buffer at 0.45 V/cm for 20 min. The slides were then dehydrated subsequently in 75% and 96% ethanol and left to fully dry at room temperature. The results were visualized under an epifluorescent microscope Leitz—Orthoplan, Vario Orthomat 2 (450/490 nm), after staining with SYBR Green (Roche Diagnostics GmbH). Treatment of CCL1 cells with 5 mM H_2_O_2_ for 30 min at 37 °C was performed as a positive control for genotoxicity. Results were analyzed by using the CometScore software. Three repetitions of the experiment have been done and data were evaluated using Excel 2016 software where values for the measured Olive Moment in all probes were presented as MEAN values ± STDEV.

#### FACS analysis for probing the cytostatic activity of the tested solvents and extracts

CCL1 cells treated for 24 h with the NADES solvents and extracts were fixed in 100% ice-cold ethanol and stored at − 20 °C overnight. The cell pellets were washed twice with 1xPBS (2.68 mM KCl, 1.47 mM KH_2_PO_4_, 1.37 mM NaCl, 8 mM Na_2_HPO_4_), pH 7. Incubation with RNase A (100 µg/ml) for 30 min at 37 °C followed. Before FACS analysis the cells were stained with propidium iodide (50 µg/ml) for 20 min at dark. FACS analysis was performed by a BD FACSCanto apparatus and the results were analyzed by FlowJo software V10.

## Results and discussion

### Extraction and evaluation of extraction efficiency

Four NADES were selected based on literature data of their polarity, close or higher than 70% ethanol. They are described in Table 1, with corresponding abbreviations, and data of their density and polarity measured with the solvatochromic dye Nile red (lower E_NR_ values mean higher polarity, [4].

**Table 1:** Composition and characteristics of the NADES

Abbreviation	Component 1	Component 2	Molar ratio	Water (%)	E_NR_ (kcal/mol)	Density (g/ml)
XXGly	Choline chloride	Glycerol	1:2	0	50.03	1.17
CAPD	Citric acid	1,2-Propanediol	1:4	0	48.87	1.19
XXGlH	Choline chloride	Glucose	5:2	30	49.17	1.16
XXPD	Choline chloride	1,2-Propanediol	1:3	0	50.69	1.06
EtOH 70%					50.87	0.88

The dry aerial parts of both selected plants were extracted with the four NADES and 70% ethanol as a reference solvent. Ultrasound assisted extraction was applied to accelerate the process, because of the significant viscosity of the NADES. The NADES choline chloride-glucose 5:2 was too viscous and to enable mass transfer, water was added to make it suitable for extraction. It is known that addition of water decreases the viscosity of NADES and weakens the hydrogen bonding interaction between its components, but dilution under 50% usually does not lead to a matrix disruption into individual components [9].

The extraction yield was evaluated by measuring the total phenolics and total flavonoids in the extracts. Extraction with 70% ethanol under the same conditions was used as a reference. The results are presented in Table 2.

**Table 2:** Amount of extracted plant constituents (percentage of dry plant material)

Extraction solvent	*S. scardica*		*P. major*	
	Total phenolics, %	Total flavonoids, %	Total phenolics, %	Total flavonoids, %
70% EtOH	5.4 ± 0.2	1.14 ± 0.02	5.55 ± 0.03	1.11 ± 0.06
XXGly	4.6 ± 0.3	1.02 ± 0.03	3.27 ± 0.2	0.51 ± 0.06
CAPD	4.2 ± 0.1	0.87 ± 0.03	3.06 ± 0.03	0.69 ± 0.06
XXGlH	6.3 ± 0.2	1.20 ± 0.06	6.99 ± 0.5	0.22 ± 0.06
XXPD	5.6 ± 0.2	1.20 ± 0.06	4.62 ± 0.2	0.90 ± 0

The results demonstrated that some of the NADES extracted more bioactive compounds than the classic water-alcohol mixture. The water-containing NADES XXGlH was the most effective one, it extracted more phenolics and more flavonoids from the mountain tea that 70% ethanol. In the case of plantain phenolics XXGlH was again the most effective solvent: it extracted 25% more phenolics, compared to the reference solvent. However, none of the four NADES extracted flavonoids from plantain more efficiently than the 70% ethanol. This difference could be explained considering the specific chemical structures of flavonoids in the two medicinal plants. In *S. scardica*, flavonoid diglycosides predominate [10], while the major flavonoids in *P. major *are flavone monoglycosides [11], which are less polar than diglycosides. As the chosen NADESs have polarities higher than 70% ethanol, the better extraction of more polar flavonoid diglycosides can be explained by this fact, at least to some extent.

### Antimicrobial activity

Recently, it became clear that NADES components can be selected not only to fine-tune solvent physicochemical characteristics but also to improve the biological activity of dissolved active compounds [12, 13]. That is why we decided to check the antimicrobial activity of the extracts, and to compare the results with the activity of ethanol extracts, based on total phenolics concentration. The minimum inhibitory concentrations (MIC) and minimum bactericidal concentrations (MBC) of total phenolics in the extracts were measured. In addition, the metabolic activity of the microorganisms after the treatment was studied by measuring the dehydrogenase activity (DEHA) with the MTT test. The results of these tests are shown in Table 3.

**Table 3:** MIC, MBC and dehydrogenase activity (MTT test) of total phenolics in different solvent extracts of *S. scardica* and *P. major*

Sample/indicator	XXGly/S^a^	XXGly/P^b^	CAPD/S^a^	CAPD/P^b^	70% EtOH/S	70% EtOH/P
*S. pyogenes* SAIM 10535						
MIC (µg/ml)	380	NA^c^	0.19	NA	> 67.5	> 69.3
DEHA (%)	–	NA	39.88 ± 9.6	NA	–	–
MBC (µg/ml)	380	NA	1.56	NA	> 67.5	> 69.3
*E. coli* ATCC 35218						
MIC (µg/ml)	47.5	38.75	0.39	3.984	> 67.5	> 69.3
DEHA (%)	–	–	1.31 ± 0.87	–	–	–
MBC (µg/ml)	47.5	38.75	0.78	3.984	> 67.5	> 69.3
*S. aureus* ATCC 29213						
MIC (µg/ml)	NA	NA	0.098	1.99	> 67.5	> 69.3
DEHA (%)	NA	NA	9.8 ± 2.3	–	–	–
MBC (µg/ml)	NA	NA	0.39	1.99	> 67.5	> 69.3
*P. aeruginosa* ATCC 27853						
MIC (µg/ml)	23.75	19.37	NA	NA	> 67.5	> 69.3
DEHA (%)	–	–	NA	NA	–	–
MBC (µg/ml)	23.75	19.37	NA	NA	> 67.5	> 69.3
*C. albicans* SAIM 562						
MIC (µg/ml)	23.75	19.37	0.19	1.99	> 67.5	> 69.3
DEHA (%)	–	–	7.595 ± 2.1	–	–	–
MBC (µg/ml)	23.75	19.37	0.78	1.99	> 67.5	> 69.3
*B. cereus* ATCC 9634						
MIC (µg/ml)	23.75	38.75	NA	NA	> 67.5	> 69.3
DEHA (%)	50 ± 12.3	–	NA	NA	–	–
MBC (µg/ml)	47.5	38.75	NA	NA	> 67.5	> 69.3

Extracts with XXPD and XXGlH are inactive against all tested microorganisms

^a^Extract of *S. scardica*

^b^Extract of *P. major*

^c^NA: no activity. DEHA data are expressed as the mean ± SD of three measurements

The most potent antimicrobial activity was observed for CAPD/S against *S. pyogenes*, *E. coli*, *S. aureus* and *C. albicans* with MICs between 0.098 and 0.39 µg/ml total phenolics. CAPD/P was active against *S. aureus*, *E. coli* and *C. albicans* (MICs were in the range 1.99–3.98 µg/ml total phenolics). The plantain extracts had MBC = MIC for the three test microorganisms against which they were active. In this case the DEHA activity cannot be determined because it is equal to zero, as there are no metabolically active cells present. The extracts XXPD/S, XXPD/P, XXGlH/S, and XXGlH/P were inactive against the tested microorganisms. Surprisingly, the extracts with the NADES which was most effective in the extraction of phenolics and flavonoids, XXGlH, showed no antimicrobial activity. This could be due to qualitative differences between the extracts obtained with different solvents, however, qualitative analysis of the extracts was not performed. The present work aimed to find the most effective NADES for extraction of *S. scardica* and *P. major* in terms of antimicrobial potential. Optimization of the extraction conditions with the most effective solvent is the subject of future work, when we intend to apply more detailed analysis of individual constituents.

In most cases where antimicrobial activity was observed, it was much higher than the one of the traditional ethanol extracts. Such antimicrobial potentiating effects of NADESs was also observed in the case of propolis NADES extracts [14]. Although the addition of more than 50% of water to NADES during the antimicrobial tests breaks the NADES supramolecular complex, the individual NADES constituents could contribute to the overall effect of the solution [15] and some synergistic effects are also to be considered. Especially in the case of the CAPD extract of *S. scardica*, the increased effect compared to the other extracts can be partly explained by the effect of the presence of citric acid as an element of the NADES. In the literature there are indications that NADES containing organic acids possess higher antimicrobial activity, since some organic acids present many pharmacological effects [16].

### Genotoxicity and cytotoxicity

Any further implication of the tested four NADES in the practice requires proofs for their genotoxicity safety. Many data in the literature show the genotoxicity of a broad range of food, cosmetic and pharmaceutical additives regardless of their nature [17–19]. Some data point to the fact that these substances added to the products consumed by or applied on people exert their genotoxicity at allowed concentrations [17, 20, 21]. This unambiguously entails the application of sensitive tests for fast and accurate evaluation of the potential of any substance that is planned to be implicated in food, pharmaceutical and cosmetic practices to induce general cytotoxicity via inhibiting cell proliferation and/or damage in DNA [22–24]. The method of Comet Assay is a brilliant technology for fast and sensitive analysis of genotoxicity [25, 26]; it requires single cells, is fast and with high precision evaluates all kinds of DNA damages. Data quantification allows precise estimation of genotoxicity. In this study, the tested NADES solvents and the extracts which demonstrated antimicrobial activity: the extracts with XXGly and CAPD of *S. scardica* and *P. major*, were tested for their genotoxicity. The preparation for this test first required the evaluation of the in vitro cytotoxicity of the tested substances. Therefore, the in vitro cytotoxicity of the NADESs and extracts was determined on the normal mouse fibroblast cell line CCL-1 (Table 4) for two exposure times—24 and 72 h. The IC_50_ values calculated after 24 h exposure time were used for performing the genotoxicity assay. The calculated IC_50_ concentrations of all tested samples are given in Table 4.

**Table 4:** Calculated IC_50_ values for the tested four NADES solvents and the extractss from *S. scardica* and *P. major*

Tested samples	Median inhibitory concentrations, parameters of the model and exposure time					
	24 h			72 h		
	IC_50_^a^ (% v/v, μl)	95% CI^b^	R	IC_50_ (% v/v, μl)	95% CI	R^c^
Solvents						
XXGly	1.180	0.985–1.412	0.904	1.394	1.261–1.542	0.950
CAPD	0.089	0.075–0.105	0.940	0.141	0.126–0.158	0.971
XXGIH	0.077	0.043–0.139	0.980	0.096	0.085–0.108	0.964
XXPD	2.677	1.516–4.611	0.906	1.503	1.197–1.887	0.707
Extracts						
XXGly/S	0.803	0.563–1.148	0.952	0.589	0.391–0.886	0.901
XXGly/P	1.267	0.957–1.679	0.912	1.094	0.845–1.418	0.894
CAPD/S	0.046	0.040–0.054	0.955	0.148	0.136–0.161	0.971
CAPD/P	0.045	0.039–0.053	0.956	0.024	0.021–0.027	0.962

	IC_50_ (µg/ml tP^d^)	95% CI	R	IC_50_ (µg/ml tP)	95% CI	R
Extracts						
XXGly/S	12.212	8.550–17.450	0.952	8.953	5.941–13.490	0.901
XXGly/P	15.711	11.863–20.820	0.912	13.566	10.470–17.590	0.894
CAPD/S	0.046	0.040–0.054	0.955	0.148	0.136–0.161	0.971
CAPD/P	4.607	4.026–5.376	0.956	0.243	0.210–0.275	0.962

^a^IC_50_: inhibitory concentration that inhibits the cell growth by 50%

^b^CI: confidence interval

^c^R: coefficient of correlation

^d^tP: total phenolics

As visible from the median inhibitory concentrations in Table 4, the solvent XXGly was less cytotoxic than CAPD. The same trend was observed for the relevant extracts. Interestingly, the cytotoxicity of the solvents XXGly, CAPD and XXGIH diminished with time and was less pronounced after 72 h of exposure than after 24 h. All extracts showed dose and time dependent cytotoxicity. The XXGly/*S* extract exhibited more potent antiproliferative effect on mouse fibroblasts than XXGly/*P* and was more cytotoxic to the cells than the solvent itself in both exposure periods. The cytotoxicity of XXGly/P was more pronounced than that of the solvent after the longer exposure period (72 h). CAPD/S showed a twofold higher antiproliferative effect on the cells after 24 h of exposure than CAPD. However, after 72 h the effect of CAPD/*S* diminished slightly compared to that of the solvent at the first incubation period. The cytotoxicity of the CAPD/*P* extract was time-dependent and the IC_50_ values were significantly lower than those of the pure solvent, especially after long exposure time. Considering the MIC values determined by the BMD assay, it can be seen that CAPD/*P* exhibited anti-staphylococcal and anti-fungal activities at concentrations 3.9 and 1.99 µg/ml tP, respectively, that are significantly lower than the median inhibitory concentration cytotoxic for normal mouse fibroblasts (IC_50_/24 h = 4.6 µg/ml tP).

After estimation of the cytotoxicity of the studied NADES solvents and extracts, CCL1 cells were subjected to Comet assay. CCL1 cells were treated with the four tested NADES and extracts of *S. scardica* and *P. major* with XXGly and CAPD, for 24 h at optimal conditions. Both the pure NADES and the NADES extracts were applied to the monolayer cells at concentrations of IC_50_, ½IC_50_ and ¼IC_50_, estimated by MTT tests (Table 4). Untreated cells were used as negative control while CCL1 cells treated for 30 min at 37 °C with 5 mM H_2_O_2_ were used as a positive control for genotoxicity.

**Fig. 1:**
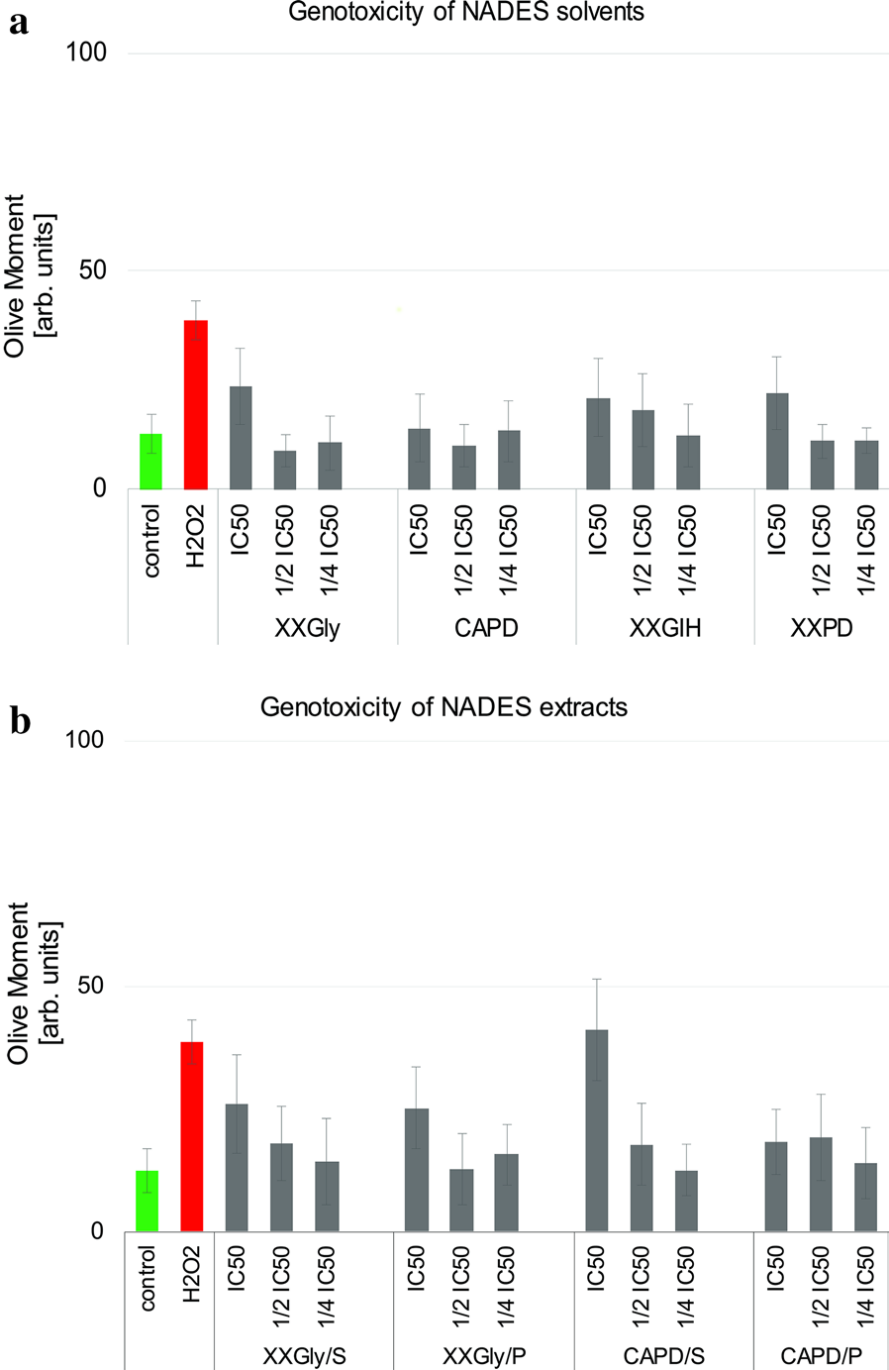
Genotoxicity of the tested NADES c solvents and extracts on CCL1 cells. **a** Tail Olive Moment of CCL1 cells treated with the tested NADES. **b** Tail Olive Moment of CCL1 cells treated with the tested NADES extracts

The Comet Assay results were quantified with the CometScore software and the values for the Olive Moment are presented in Fig. 1. Results demonstrated that all NADESs at a concentration of IC_50_ showed moderate to subtle genotoxicity effect except XXGly that at a concentration of IC_50_ demonstrated the highest genotoxic potential in comparison to all other solvents applied at IC_50_. Dilution two and four times of the applied IC_50_ of all solvents showed lack of genotoxic potential on the tested CCL1 cells. The less genotoxic were all tested concentrations of CAPD (Fig. 1a). Further, extracts of *S. scardica* and *P. major* with XXGly and CAPD were tested for genotoxicity with the method of Comet Assay and results are shown in Fig. 1b. All extracts applied at concentration of IC_50_ demonstrated genotoxicity on CCL1 cells, especially CAPD/S. Specifically, XXGly/S and XXGly/P, CAPD/S and CAPD/P showed a decrease in the detected genotoxicity when applied at ½ and ¼ of IC_50_. The most harmless was CAPD/P.

These NADES probes demonstrated a lack of genotoxicity at all tested concentrations. Conserning the detected genotoxicity the NADES solvents can be classified in a row showing an increase in genotoxicity with an increase in the tested concentrations of the compounds. The NADES solvents and extracts are listed in the direction left to right, which marks increased genotoxicity. The NADES solvents are arranged as follows: CAPD < XXGIH < XXPD < XXGly. The extracts can be classified like this: CAPD/P < XXGly/P < XXGly/S < CAPD/S.

**Fig. 2:**
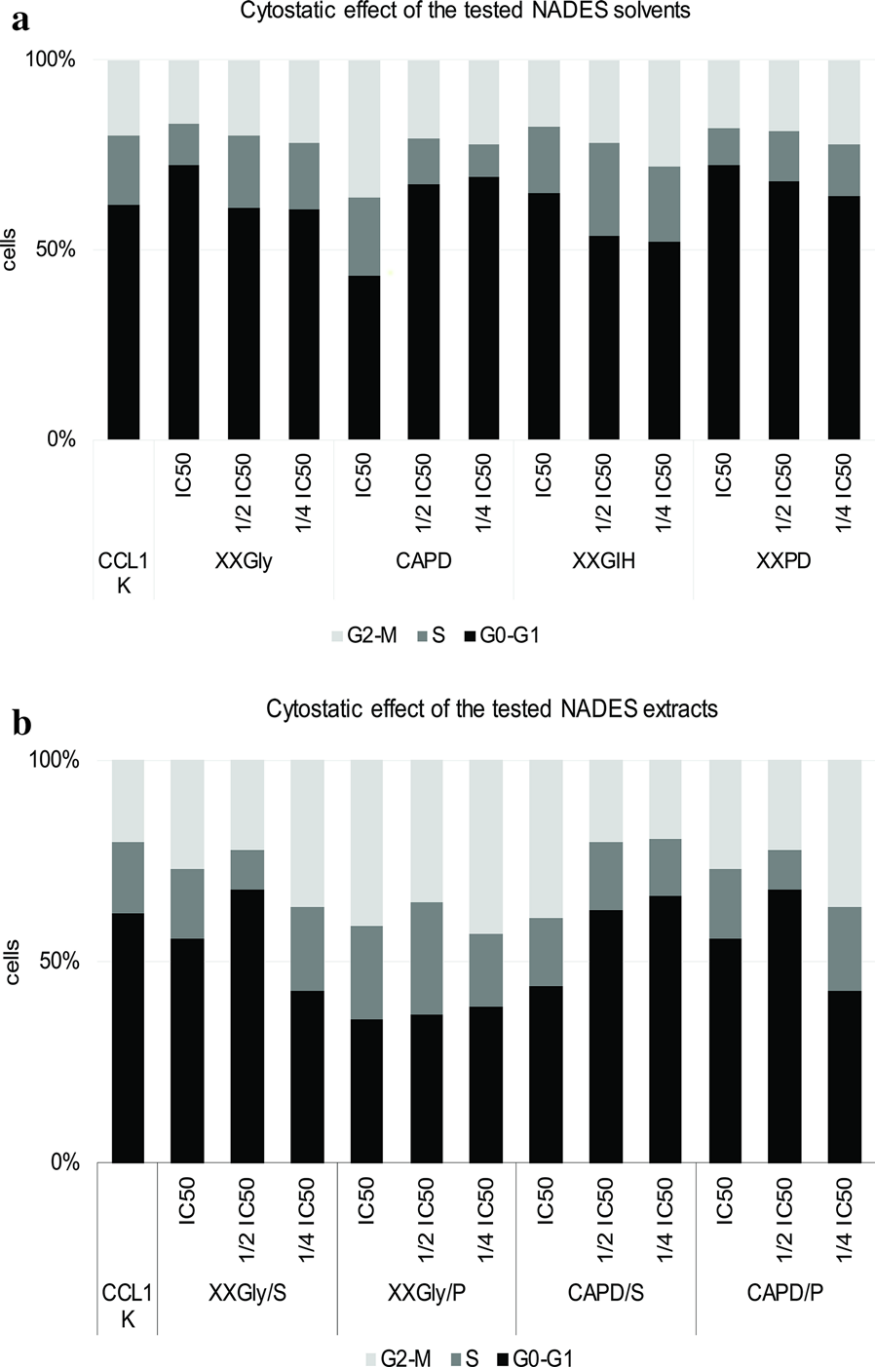
Cell cycle evaluation using FACS of CCL1 cells treated with the tested NADES solvents and extracts. **a** Distribution of cells through the cell cycle phases in CCL1 cells treated with NADES probed on CCL1 by FACS cells after 24 h of treatment. **b** Distribution of cells through the cell cycle phases in CCL1 cells treated with NADES extracts

Generally, all tested NADES revealed little changes in the distribution of cells in the different phases of the cell cycle after treatment for 24 h with increasing concentrations. The closer look at the graph in Fig. 2a shows that XXGIH at all tested concentrations, most explicitly at ½ and ¼IC_50_ demonstrated decrease in the population of cells in G0/G1, suggesting a slight cytostatic effect. CCL1 cells treated with CAPD/S applied at IC_50_, and CAPD/P at all tested concentrations, especially at ¼IC_50_ showed fewer cells in G0/G1. These results correspond with the detected genotoxicity in the samples. The detected genotoxicity led to moderate cytostatic effect on the CCL1 cells.

All tested NADES proved harmless genome integrity of the tested CCL1 cells. The detected little changes in the percentage of cells in different phases of the cell cycle can be due to cell culture asynchronization.

## Conclusions

In conclusion, our results confirm the promising potential of NADESs as solvents for extraction of the biologically active constituents of popular medicinal plants and confirm the suggestion that NADES can improve the biological effects of bioactive extracts [12]. Best extraction results for total phenolics for the studied plants were obtained using XXGlH, but surprisingly these extracts were inactive against all tested microorganisms and were not subjected to further studies of in vitro cytotoxic and genotoxic activity. The most effective were the extracts with CAPD. The presence of citric acid and some synergistic effects with *Sideritis* constituents may play a role, as CAPD extract of *S. scardica* was much more active compared to the respective *P. major* extract.

The use of NADESs allows avoiding organic solvents and the significant antimicrobial potential of the NADES extracts combined with the low toxicity and genotoxicity of the solvents and the extracts present a very promising perspective for using these extracts in food, cosmetic and pharmaceutical formulations. Of course, further studies are needed to answer the question of the influence of the NADES on the bioactivity of the dissolved substances, and to elucidate the fine molecular mechanisms of their action in model cells and organisms.

## Data Availability

All materials used in the present study are mentioned in “Materials and methods” section and the data will be available upon request.

## Abbreviations

ATCC: American Type Cell Culture Collection
BHIB: Brain Heart Infusion Broth
BHIA: Brain Heart Infusion Agar
CAPD: Citric acid-1,2-propanediol 1:4
DEHA: Dehydrogenase activity
DES: Deep eutectic solvent(s)
FACS: Fluorescence activated cell sorting
MBC: Minimum bactericidal concentration
MHA: Muller Hinton Agar
MHB: Muller Hinton Broth
MIC: Minimum inhibitory concentration
MTT: 3-(4,5-Dimethylthiazol-2-yl)-2,5-diphenyltetrazolium bromide
NADES: Natural deep eutectic solvent(s)
tP: Total phenolics
XXGlH: Choline chloride-glucose 5:2 and 30% water
XXGly: Choline chloride-glycerol 1:2
XXPD: Choline chloride-1,2-propanediol 1:3
/P: Extract of *Plantago major*
/S: Extract of *Sideritis scardica*
